# Study on the High-Efficiency Preparation of Superhydrophobic Polymer Thin Films by Continuous Micro/Nano Imprinting

**DOI:** 10.3390/polym16070912

**Published:** 2024-03-26

**Authors:** Zhi Chen, Yumeng Wei, Cheng Wu, Guojun Zhang, Fenglin Han

**Affiliations:** 1 State Key Laboratory of Precision Manufacturing for Extreme Service Performance, College of Mechanical and Electrical Engineering, Central South University, Changsha 410083, China; 217052@csu.edu.cn (Z.C.); yumengw@csu.edu.cn (Y.W.); w18358838436@163.com (C.W.); 2 Guangdong Provincial Key Laboratory of Manufacturing Equipment Digitization, Guangdong HUST Industrial Technology Research Institute, Dongguan 523808, China; 18202764498@163.com

**Keywords:** superhydrophobic polymer thin film, micro/nano imprinting, forming mechanism, rheological filling model

## Abstract

In order to improve the preparation efficiency, quality stability, and large-area preparation of superhydrophobic thin films, a roll-to-roll continuous micro–nano imprinting method for the efficient preparation of superhydrophobic polymer films is proposed. A wear-resistant mold roller with hierarchical microstructure is prepared by wire electrical discharge machining (WEDM). The rheological filling model is constructed for revealing the forming mechanism of superhydrophobic polymer films during continuous micro/nano imprinting. The effects of imprinting temperature, rolling speed and the surface texture size of the template on the surface texture formation rate of polymer films are analyzed. The experimental results show that, compared with other process methods, the template processed by WEDM shows excellent wear resistance. Moreover, the optimal micro/nano imprinting parameters are the mold temperature of 190 °C (corresponding film temperature of 85 ± 5 °C), rolling speed of 3 rpm and roller gap of 0.1 mm. The maximum contact angle of the polymer film is 154°. In addition, the superhydrophobic polymer thin film has been proven to have good self-cleaning and anti-icing performance.

## 1. Introduction

The surface of the lotus leaf is composed of micron-sized papillae and nanoscale wax crystals, which make it exhibit an excellent self-cleaning property [1]. Solar batteries work outdoors for a long time, and the surface of the battery plate accumulates dust easily, which seriously affects the power generation efficiency of solar cells. If a transparent self-cleaning superhydrophobic film is coated on the surface of the solar battery, the dust can be taken away by rainwater. Similarly, the application of this film to ceramics, fabrics, vehicles and other things exposed to the environment can reduce cleaning costs and improve convenience of use.

Inspired by the biological superhydrophobic surfaces in nature, researchers have proposed many methods for preparing superhydrophobic polymer films. Polymer thin film surface can become a superhydrophobic surface through constructing a rough surface and reducing surface energy. This is because the micro-scale and nano-scale secondary structures on the rough surface increase the surface area of the sample. Air is stored between the pits and micropores, and an air layer is formed between the sample and the water droplets, so it has superhydrophobic properties. The common preparation methods for superhydrophobic polymer films mainly include UV nano imprinting, phase separation, chemical deposition method, etc. [2,3,4].

UV nano imprinting was a commonly used method for preparing superhydrophobic polymer films [5,6]. Firstly, a layer of photoresist with good UV sensitivity and flowability was applied to the base. The photoresist formed a copying structure under the binding of a certain pressure transparent template. The photoresist was cured and formed under UV irradiation. Yao et al. [7] used polydimethylsiloxane (PDMS) to replicate the micro/nano structure on the surface of lotus leaves. And then, the PDMS film was treated as a negative mode. Fluoropolyacrylate (FPAM) thin films were prepared using UV nano imprinting technology. The prepared film had a maximum contact angle of 172° and excellent superhydrophobicity and superhydrophilicity. However, the preparation cost of UV nano imprinting is relatively high. The phase separation method is a simple method for preparing porous superhydrophobic polymer films [8,9]. Polymer solutions can precipitate solid polymer solutes under specific induction conditions. Wang et al. [10] prepared superhydrophobic membrane using the water-assisted thermally induced phase separation method. The prepared membrane surface had a rough honeycomb microstructure and strong oil–water separation ability. The adsorption capacity of the membrane was 27.3 times its own weight, which could be used as an efficient filtration membrane. The phase separation method for preparing superhydrophobic thin films has limited material types and low preparation efficiency, making it unsuitable for the preparation of large-area superhydrophobic thin films. The chemical deposition method utilizes one or more vapor phase compounds or substances to undergo chemical reactions on the substrate surface for generating thin films. Shi et al. [11] prepared micron-sized rectangular columns on the polyimide surface using a combining method of reactive ion etching and plasma chemical vapor deposition. The prepared film had good superhydrophobicity and anti-icing property. The chemical deposition method is unsuitable for the preparation of large-area superhydrophobic polymer films due to the complex preparation and high cost.

Template imprinting is a method of copying and transferring microstructure patterns from the template to the target surface [12,13]. Zhang et al. [14], Kim et al. [15] and Moon et al. [16] prepared microscale array structures on the polymer surface using the hot embossing method. After the chemical modification with nanoparticles, the micro/nano hierarchical structures were formed on the polymer surface. The prepared superhydrophobic film had excellent self-cleaning performance. The template imprinting method has several advantages, including a simple preparation process, low cost and high repeatability, which make it the preferred method for preparing large-area superhydrophobic polymer films. However, when the planar template is used to prepare superhydrophobic polymer films, there is the problem of low preparation efficiency. Moreover, the single-stage micro-nano structure on the surface of the template is easily damaged during the demolding process and after repeated use, which greatly reduces the service life span of the template. The temperature-dependent viscoelasticity characteristics of polymer make the micro/nano imprinting process more complex. It is difficult to obtain the optimal micro–nano imprinting parameters. The material of this micro–nano imprinting experiment is linear low-density polyethylene (LLDPE). The prepared polymer film exhibits durability. Zhang et al. [17] prepared a flexible superhydrophobic/superoleophilic film based on low density polyethylene (LDPE)/ethylene propylene diene monomer (EPDM) thermoplastic vulcanizate (TPV) by the sandpaper template method. The film can be easily rolled up and loaded on the valve. Under appropriate pressure, the oil can be completely separated from the oil–water mixture through the film. Tseng et al. [18] and Xu et al. [19] prepared a graphene layer on the surface of polyimide film using the biological template and laser etching method. By adjusting the process parameters of laser etching, good superhydrophobicity of the polymer film could be achieved, which had important application prospects in anti-sweat and serum adhesion. Laser fabrication is found to be one of the best techniques for the fabrication of superhydrophobic surfaces.

In traditional template methods, the preparation efficiency of flat templates is low and not suitable for large-scale preparation. When the roll-to-roll template method is adopted, although it can prepare superhydrophobic films on a large area, the wear of mold rollers and demolding defects significantly increase the preparation cost and reduce the quality stability. To address the above issues, this paper first proposes the WEDM method to fabricate a hierarchical microstructure on a roller for obtaining a wear-resistant superhydrophobic template. Through continuous pulse discharge, wire electrical discharge machining forms an instantaneous high-temperature and high-pressure effect on the surface of the workpiece, so that the material on the surface of the workpiece is eroded due to melting or vaporization, forming a large number of micro–nano-scale pits/bulges [20,21], thus creating a superhydrophobic rough surface. The hierarchical microstructure composed of surface texture and discharge morphology could not only obtain superhydrophobicity, but also improve the service life of mold rollers and reduce demolding defects.

In order to improve the preparation efficiency, quality stability, and large-area preparation of superhydrophobic thin films, the method of roll-to-roll micro/nano imprinting is adopted to high-efficiency preparation of superhydrophobic polymer films. Through theoretical simulation and experimental methods, the rheological filling mechanism of polymer during continuous micro/nano imprinting is explored to optimize the parameters of continuous micro/nano imprinting. A rheological filling model of polymer is established during the continuous micro–nano imprinting process. The effects of the imprinting temperature, rolling speed and surface texture size of the template on the surface texture molding rate of polymer films are analyzed so as to obtain the best micro/nano imprinting parameters. Through experimental methods, the surface texture size and micromorphology of template and polymer films are characterized. The contact angle, self-cleaning performance and anti-icing performance of the polymer film are tested.

## 2. Materials and Methods

### 2.1. Material

(1) The material of imprinting template

The material of the imprinting template is 302 stainless steel, which is an austenitic stainless steel with excellent physical and chemical properties, such as plasticity, toughness, processing performance and corrosion resistance. It is widely used in some high-end fields, including molds, aerospace, automotive manufacturing and chemical engineering.

(2) The material of polymer film

The material of polymer film is low-density polyethylene (LLDPE, LL0220AA) (Dongguan Haike Plastics, Dongguan, China). Low-density polyethylene has good low-temperature resistance, electrical insulation, transparency and processability [22], with a melting point of 110~115 °C and a processing temperature of 150~210 °C, which meets the durability requirements of polymer films. Its physical parameters are shown in Table 1.

(3) Low-surface energy coating

In order to further improve the contact angle, nano silica coating is used to reduce the surface energy of the polyethylene film. The silica coating consists of nano silica powder, polydimethylsiloxane (PDMS), curing agent (Sylgard 184) and butyl acetate solution (analytical pure, mass fraction ≥ 99.5%) (Shanghai McLean Biochemical Technology Co., Ltd., Shanghai, China). The physical parameters of nano silica are shown in Table 2.

The preparation method of SiO_2_-PDMS/butyl acetate dispersion suspension is as follows: a certain amount of PDMS (with a mass ratio of precursor and curing agent of 10:1) is mixed with butyl acetate solution. The mixing solution is ultrasonic stirred at 30 °C for 15 min. Then, a certain amount of hydrophobic nano silica powder is poured into the mixing solution. The suspension solution is ultrasonic stirred at 30 °C for 15 min. The composition design of the SiO_2_-PDMS/butyl acetate dispersion suspension is shown in Table 3.

**Figure 1:**
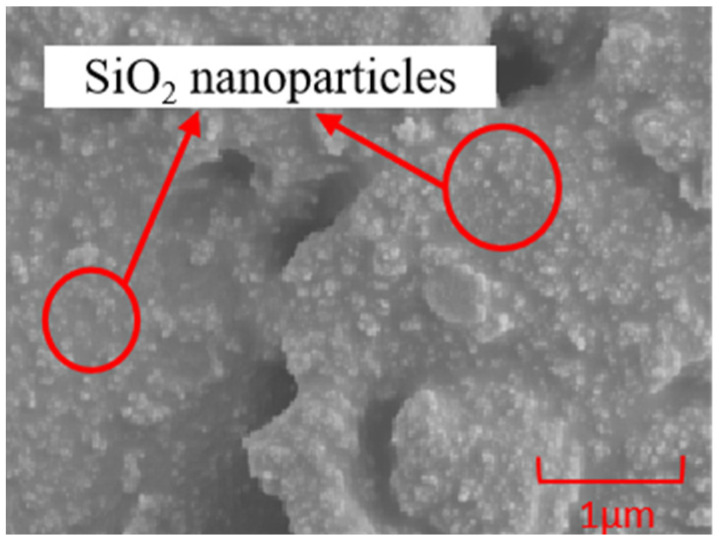
The SEM image of SiO_2_/PDMS coating.

Figure 1 shows the SEM image of the SiO_2_/PDMS coating. The crystal form of nano-silica particles is spherical and their size is between tens of nanometers and hundreds of nanometers. They are densely and evenly distributed on the PDMS substrate, which is beneficial to the improvement of the hydrophobicity of the polymer film surface.

### 2.2. Preparation Method

(1) Imprinting template

**Figure 2:**
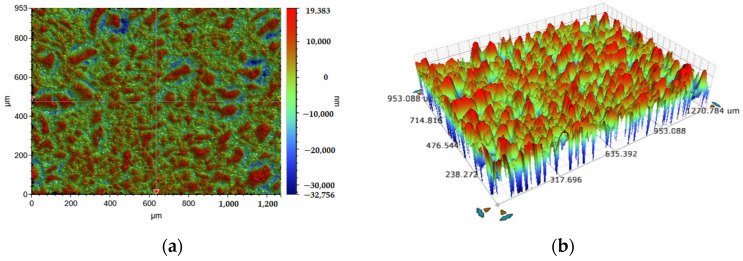
Three-dimensional surface topography of the sample after wire electrical discharge machining. (**a**) The top view of the sample surface. (**b**) The equidistant view of the sample surface.

**Figure 3:**
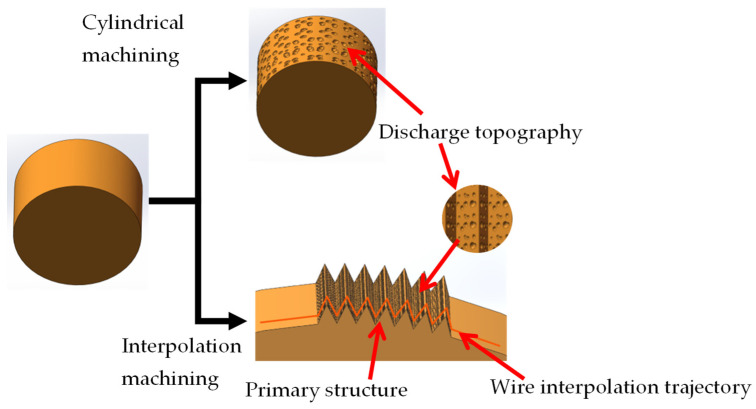
The preparation principle of hierarchical microstructures by WEDM.

In order to improve the wear resistance and service life of the imprinting template, this paper proposes WEDM (AccuteX AU-3001, Taiwan, China) to prepare superhydrophobic templates. According to the principle of electrical discharge machining, the workpiece surface swept by the wire electrode will naturally form discharge morphology, including discharge pits and protrusions (0.1–20 μm). A set of discharge parameters is selected to maximize the apparent contact angle of the workpiece: peak current of 10 A, servo voltage of 80 V, pulse on time of 400 ns, pulse off time of 150 ns, wire tension of 2400 g and feed speed of 10 mm^2^/min. The dielectric is deionized water. The wire electrode is brass wire with a diameter of 0.25 mm. Figure 2 shows the three-dimensional surface morphology of the plane processed by this set of processing parameters. The surface roughness of the plane sample is 5.4 μm, and the surface bulge is dense and uniform. WEDM can fabricate many types of surface textures, such as semicircle, rectangle, trapezoid and triangle, etc. Considering the convenience of demolding, the isosceles triangular texture is chosen as the surface texture of the superhydrophobic template. Figure 3 shows the schematic diagram of the principle of constructing hierarchical microstructures by WEDM, which consists of primary structure (surface texture) and secondary structure (discharge morphology). Through the imputation motion of a wire electrode, the surface texture (100–500 μm) can be fabricated on the template [23], which can improve the hydrophobic properties of the template, thereby obtaining a superhydrophobic template. The experiment of WEDM is completed on an Accutex AU-3001 (Taiwan, China) low-speed WEDM machine tool.

The surface of the roller template is evenly divided into eight parts. Eight sets of isosceles triangular surface textures are manufactured separately in each part. The structural parameters include the bottom edge length (l) and height (h) of the triangular surface texture. The parameter design of the triangular surface texture on the roller template surface is shown in Table 4.

(2) Polymer film

**Figure 4:**
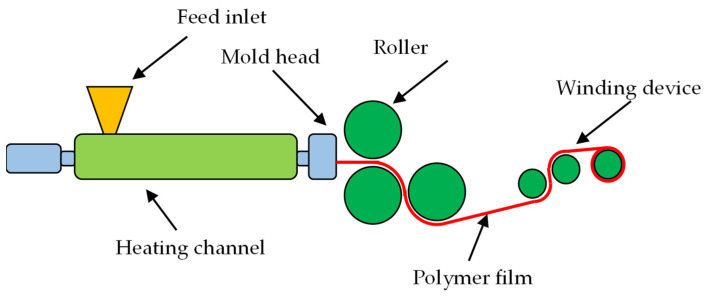
Schematic diagram of continuous micro/nano imprinting for preparing polymer thin films.

The continuous micro/nano imprinting equipment for preparing polymer thin films is shown in Figure 4, mainly including the feed inlet, heating channel, mold head, roller and winding device. The polymer particles enter the heating channel through the feed inlet. Under the heating effect of electromagnetic induction, the melted polymer is squeezed into the mold head by the screw. The polymer produced from the mold head is a high-temperature and flat film. By continuous micro/nano imprinting, the microstructure on the roller is replicated onto the polymer film. A cooling water channel is installed in the lower roller, which can quickly reduce the temperature of the polymer film. The winding device provides winding power, allowing continuous micro/nano imprinting to continue.

### 2.3. Test Method

(1) Micromorphology

Scanning electron microscopy (SEM, MIRA3LMU, Brno, Czech Republic) is chosen to observe the surface microstructure of templates and polymer films. The magnification is 500×, 2000× and 5000×.

(2) Surface roughness

Laser confocal microscopy (LSM700, Zeiss, Jena, Germany) is chosen to observe the surface morphology and surface roughness of the sample.

(3) Surface texture contour

Ultra depth of field optical microscopy (VHX-500, Keyence, Osaka, Japan) is used to measure the size of the primary surface texture of templates and polymer films.

(4) Contact angle

The high-temperature contact angle-measuring instrument (Theta, Biolin, Helsinki, Finland) is used to measure the surface contact angle of the sample. The volume of measuring water droplets is 4 μL. The contact angles of 3–5 positions on the same sample are measured. The average of multiple measurement results is taken as the final value of the surface contact angle of the sample.

(5) Wear resistance

**Figure 5:**
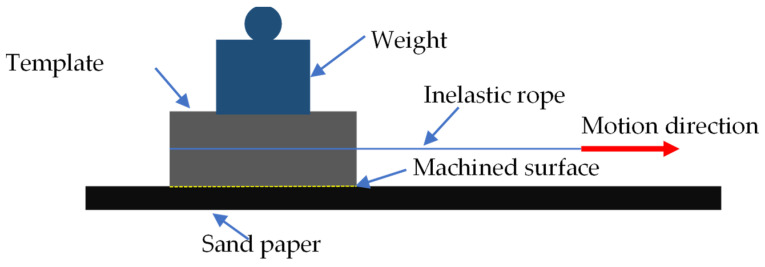
The schematic diagram of the wear test on superhydrophobic sample.

Figure 5 shows the schematic diagram of the wear test superhydrophobic sample. The sample is placed on 240 # sandpaper, and the side with triangular surface texture is in contact with the rough surface of sandpaper. The 50 g weight is pressed on the sample, and the workpiece is pulled at a constant speed by a non-elastic wire rope. In one sliding test, the moving distance of the sample is 50 mm. The sample is ground 250 times by this method. After 50 times of grinding, the size of the surface texture on the sample is measured by the ultra-depth-of-field optical microscope (VHX-500, Keyence, Osaka, Japan), and the contact angle of the sample is measured by the high-temperature contact angle measuring instrument (Theta, Biolin, Helsinki, Finland). Finally, the relationship between the grinding amount and the contact angle on the sample is obtained.

(6) Self-cleaning performance

The polymer film is tilted at a certain angle. A layer of blue dust is randomly covered on the surface of the polymer film. Subsequently, water droplets are dropped onto the surface of the polymer film using a dropper. The residual situation of water droplets and blue dust is observed to evaluate the self-cleaning performance of polymer films.

(7) Anti-icing performance

The polymer film is placed in a low-temperature test chamber. The temperature inside the chamber is adjusted to −14 °C and kept constant. Water droplets of the same size are dropped onto the surface of the polymer film. The time of starting and complete freezing is recorded through camera observation. Ice formation experiments are conducted 3–5 times on each group of polymer films. The average starting and complete freezing times are taken as the final values for the sample.

## 3. Results

### 3.1. Template Roller

#### 3.1.1. Surface Texture

**Figure 6:**
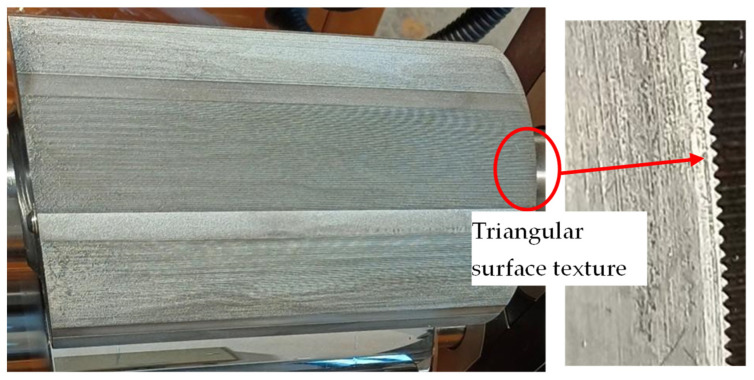
The actual image of the mold roller.

Figure 6 shows the actual image of the mold roller. Table 5 shows the actual structural size of the triangular surface texture on the mold roller. It can be seen that there is a small difference between the bottom edge lengths of the designed surface texture and the actual surface texture. However, the height of the designed surface texture is higher than that of the actual surface texture. This phenomenon is mainly due to the fact that corner errors form at the sharp corner because of the bending deformation of the wire electrode and tip discharge during the process of WEDM [24].

#### 3.1.2. Surface Micromorphology

**Figure 7:**
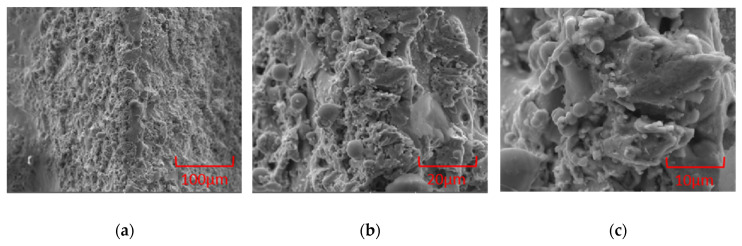
The SEM result of the surface microtopography on the template roller. (**a**) 500×. (**b**) 2000×. (**c**) 5000×.

Figure 7 shows the SEM result of the surface microtopography on the template roller. It can be seen that the workpiece surface machined by WEDM is not smooth, with many anisotropic micro/submicron pits and protrusions distributed on the surface. These discharge morphologies can provide basic conditions for the preparation of hierarchical microstructures. After applying voltage between the workpiece and the wire electrode, the dielectric between the electrode and the workpiece will be broken down when their distance is less than a certain distance. Due to the acceleration of the electric field, the electrons on the electrode surface can bombard workpiece surface without obstruction, resulting in the formation of a discharge crater by melting and vaporizing. The diameter of the single discharge crater is very small, approximately 1–50 μm. In the process of EDM, hundreds to thousands of discharge sparks are generated every second. Due to the randomness of discharge points, a large number of discharge craters are randomly generated on the workpiece surface. Most of the melted workpiece material will be carried away by the dielectric. A small portion of the melted workpiece material will reattach to the workpiece surface, forming a recast layer [25]. The shapes of the recast layer include block, spherical and coral.

#### 3.1.3. Wear Resistance

**Figure 8:**
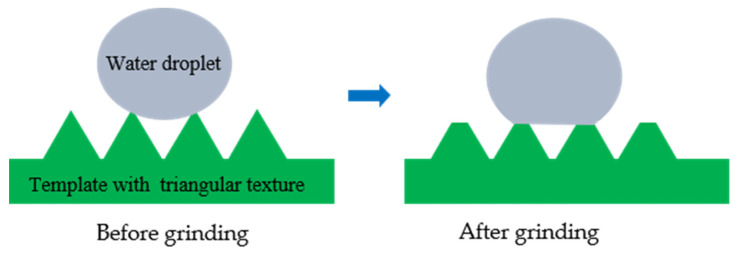
Comparison diagram before and after sample wear.

**Figure 9:**
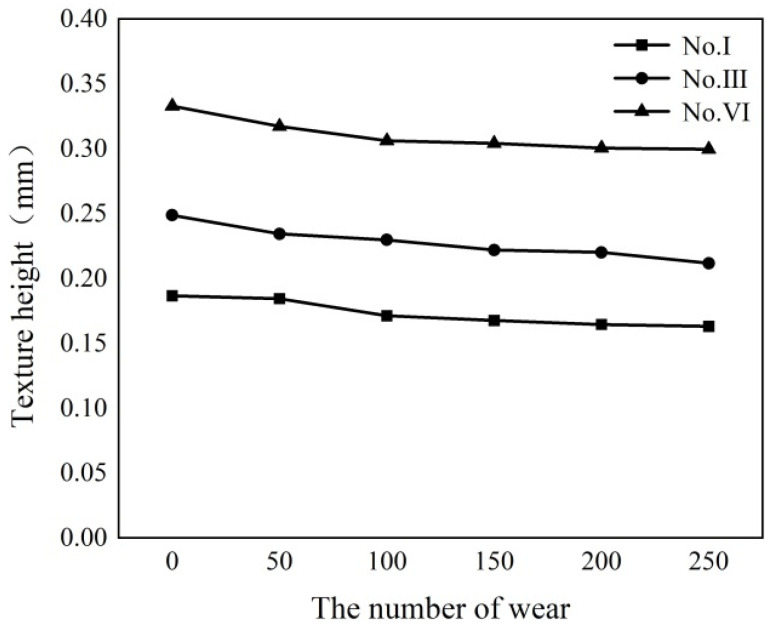
The relationship between the number of surface wear and the texture height of the sample.

Figure 8 shows the shape of the triangular texture on the template surface before and after 250 times of grinding. As shown in Figure 8, the triangular texture on the sample surface will become a trapezoidal texture after grinding. Grinding can destroy the discharge topography at the top of the triangular surface texture, but it cannot destroy the microstructure on the side of the triangular texture. As the amount of wear increases, the width of the upper edge of the surface texture increases. Therefore, as the number of grinding times increases, the surface texture height becomes smaller. After 250 grinding tests, the height difference between the original surface texture and the worn surface texture is 0.023–0.37 mm, as shown in Figure 9.

**Figure 10:**
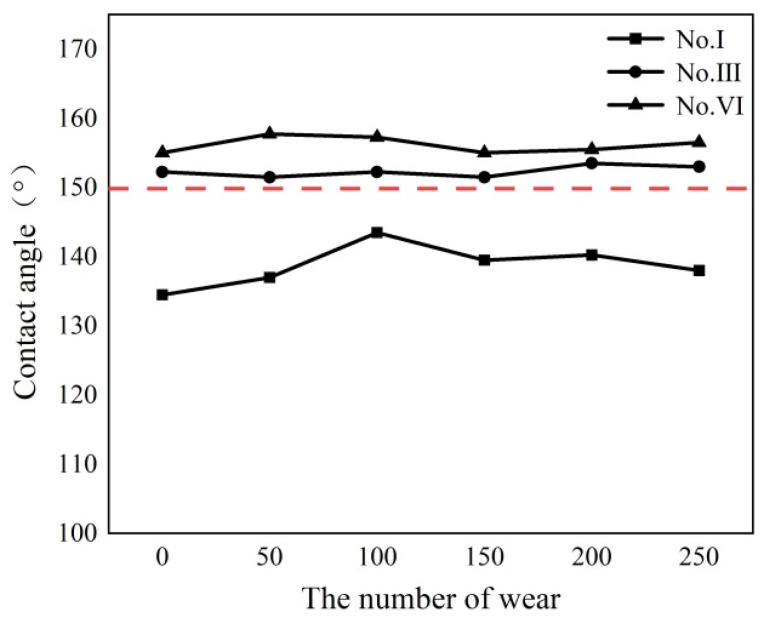
The relationship between the surface wear and the contact angle of the sample. (Where, the red dotted line is a 150° contact angle line).

**Figure 11:**
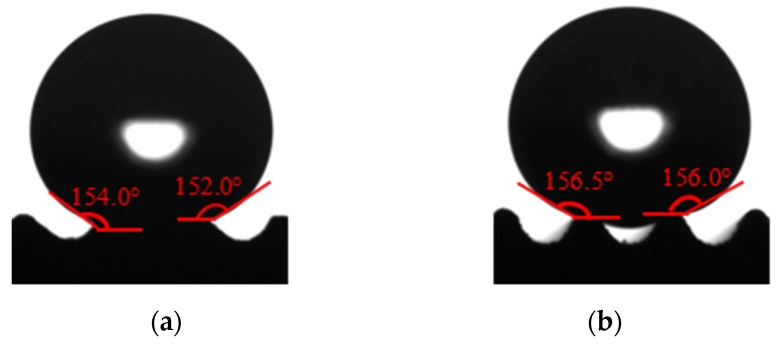
The contact angle measurement diagram of the superhydrophobic sample after 350 wear tests. (**a**) Sample III. (**b**) Sample VI.

Figure 10 shows the contact angle change of the template surface after grinding 50 times. After 250 times of grinding, the contact angle on the template surface of the superhydrophobic sample III and VI increased by 0.75–1.5°. As shown in Figure 11, the final contact angles of sample III and VI are 153° and 156.5°, respectively. These two samples can still maintain the superhydrophobic state. As shown in Figure 11, due to the decreasing of the texture height of sample III, the wetting state of water droplets on the sample surface is the Wenzel state, while the wetting state of water droplets on the surface of sample VI is still the Cassie–Baxter state, and the water droplets do not penetrate the bottom of the texture.

Table 6 shows the wear resistance of superhydrophobic samples prepared by different methods. Compared with the superhydrophobic samples prepared by other methods, the templates processed by WEDM can still maintain superhydrophobic properties under the rougher sandpaper, the higher pressure and the longer grinding length. This is mainly because the triangular texture on the sample surface will become a trapezoidal texture after grinding. Grinding can destroy the discharge topography at the top of the triangular surface texture, but it cannot destroy the microstructure on the side of the triangular texture. Therefore, the superhydrophobic surface with a hierarchical structure prepared by WEDM on metal-based materials has good wear resistance and good application prospects in practical engineering.

### 3.2. Polymer Film

#### 3.2.1. The Rheological Filling Model of Continuous Micro/Nano Imprinting

**Figure 12:**
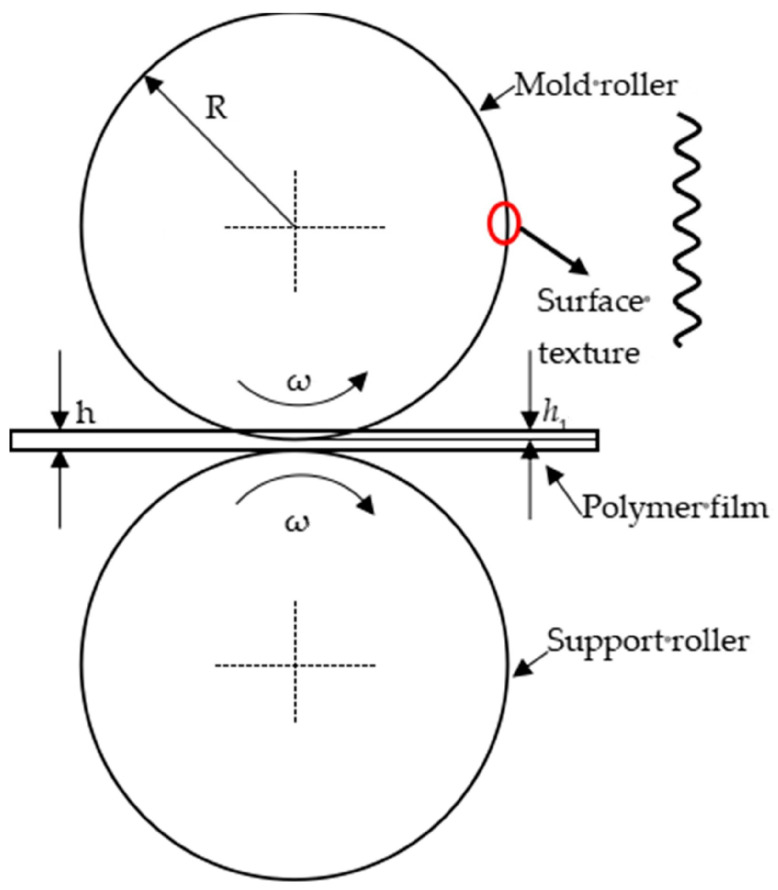
The schematic diagram of the formation of continuous micro/nano imprinted polymer films.

Figure 12 shows the schematic diagram of the formation of continuous micro/nano imprinted polymer films, mainly including the mold roller, polymer film and support roller. The radius of the mold roller is the same as that of the support roller. Both the mold roller and support roller are pure rolling at the rotational speed of ω. Triangular surface texture exists on the surface of the mold roller. The thickness of the polymer film is h. During the micro/nano imprinting process, the mold roller applies a pre-imprint (h1) on the surface of the polymer film. The polymer film moves uniformly in a straight line under the traction of the winding roller.

In the process of continuous micro/nano imprinting of a polymer film, the polymer at high temperature exhibits both solid elasticity and fluid viscosity, known as viscoelastic characteristics. This study selects the generalized Maxwell model as the constitutive model of polymers, which is used to describe the rheological properties of polymer materials. The creep form of polymer materials is shown in Equation (1):

(1) $$E(t)=E_{\infty }+\sum_{i=1}^{n}E_{i}\exp(-\frac{t}{\tau_{i}})$$

where, *E*_∞_ is the equilibrium modulus of the polymer material. *E_i_* is the corresponding relaxation modulus, *τ_i_* is the corresponding relaxation time.

The relationship between elastic relaxation modulus *E*(*t*), shear relaxation modulus *G*(*t*) and bulk relaxation modulus *K*_(*t*)_ is shown in Equations (2) and (3):

(2) $$G(t)=\frac{E(t)}{2(1+\mu )}$$

(3) $$K(t)=\frac{E(t)}{3(1-2\mu )}$$

where the elastic relaxation modulus *E*(*t*) can be determined by stress relaxation experiments. *μ* is the Poisson’s ratio of polymer materials. The Prony series forms of shear relaxation modulus *G*(*t*) and bulk relaxation modulus *K*(*t*) are shown in Equations (4) and (5):

(4) $$G(t)=G_{\infty }+\sum_{i=1}^{n_{G}}G_{i}\exp(-\frac{t}{\tau_{i}^{G}})$$

(5) $$K(t)=K_{\infty }+\sum_{i=1}^{n_{K}}K_{i}\exp(-\frac{t}{\tau_{i}^{K}})$$

where *G*_∞_ and *K*_∞_ are the final shear relaxation modulus and bulk relaxation modulus of the polymer material, respectively. $\tau_{i}^{G}$ and $\tau_{i}^{K}$ are the relaxation time. *τ_i_* = $\tau_{i}^{G}$ = $\tau_{i}^{K}$.

In stress relaxation experiments, the relaxation modulus data at a certain temperature can be obtained. According to the relationship between shear relaxation modulus, bulk relaxation modulus and elastic relaxation modulus, the Prony series coefficient of *G_i_* and *K_i_* can be obtained.

The continuous micro/nano imprinting process includes pre-imprinting, rolling and demolding. After the pre-imprinting, rolling and demolding of the mold roller, the hierarchical microstructure on the mold roller can be replicated onto the surface of the polymer film. By investigating the effects of temperature, rolling speed and the surface texture size on the texture morphology and height mold rate of triangular surfaces, the optimal micro/nano imprinting parameters and mold surface texture size are obtained. 

**Figure 13:**
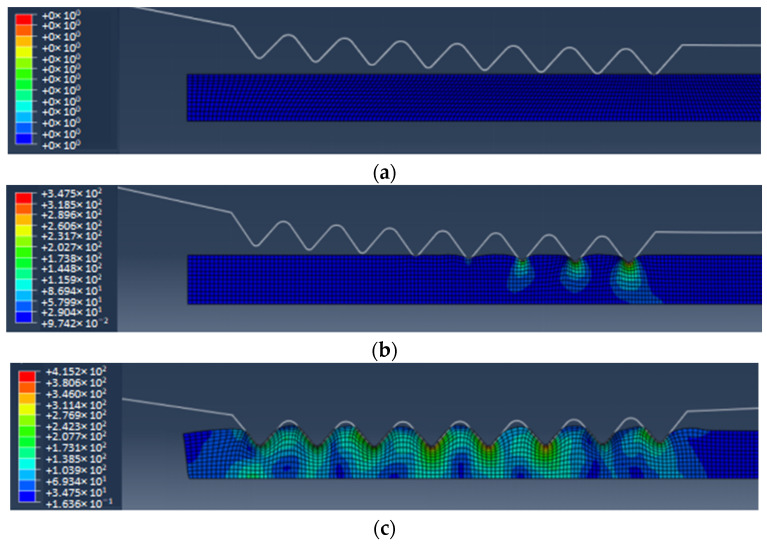

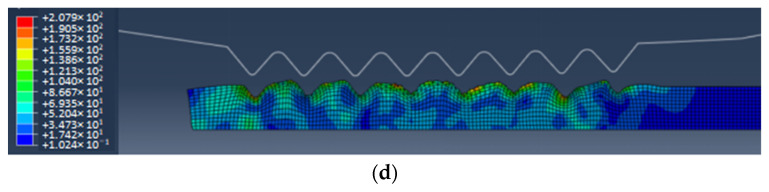
The stress field of continuous micro-nano imprinting polymer film. (**a**) Initial state. (**b**) Pre-imprinting. (**c**) Rolling. (**d**) Demolding.

Figure 13 shows the stress field of continuous micro–nano imprinting polymer film. When the mold roller is pre-imprinted on the polymer film, the surface of the polymer film undergoes slight deformation, as shown in Figure 13b. During the rolling process, the stress of the polymer film in the contact part with the top of the triangular texture of the mold roller is the largest, and the polymer is squeezed and filled into the gap of the triangular texture of the mold roller, forming a triangular microtexture on the surface of the polymer film. When the mold roller is completely imprinted into the polymer film, most of the space between the mold roller and the polymer film is filled with polymer material, as shown in Figure 13c. 

**Figure 14:**
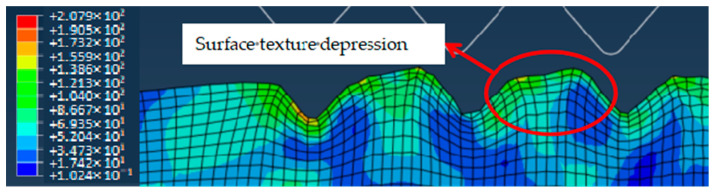
The surface texture depression of polymer film.

When the mold roller leaves the polymer film, both sides of the triangular texture in contact with the roller are cooled first; meanwhile, the corner area away from the mold roller is not cooled completely, which has both solid elasticity and fluid viscosity, resulting in its rebounding downward, thus forming a depression in the middle of the triangular texture, as shown in Figure 14. Moreover, the forming rate of the polymer surface texture decreases. After completing demolding, since the roller no longer exerts external force on the polymer film, the stress is concentrated in the place where the deformation is the largest, such as the bottom and top corners of the triangular texture.

(1) The effect of imprinting temperature on the height-molding rate of polymer film

**Figure 15:**
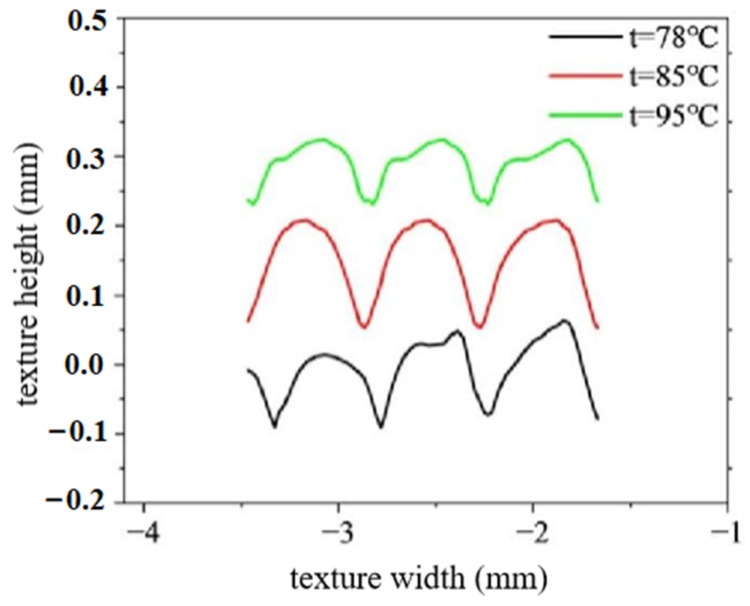
The texture shape under different imprinting temperatures.

In this subsection, the effect of imprinting temperature on the height-molding rate of polymer film is investigated, as shown in Figure 15. The imprinting temperatures are 78 °C, 85 °C and 95 °C, respectively. The other parameters remain unchanged. It can be seen that, under different imprinting temperatures, there is little difference in the width of the surface texture of polymer films. But, there is a significant difference in the height of surface texture of polymer films. The surface texture height of polymer films increases first and then decreases with an increase in imprinting temperature. Specifically, when the imprinting temperatures are 78 °C, 85 °C and 95 °C, the average height of surface texture is 0.131 mm, 0.161 mm and 0.097 mm, respectively. This phenomenon is mainly due to the following: when the temperature is low, the fluidity of the polymer material is poor, which results in the triangular surface texture of the mold roller not being filled enough. Polymer material has good flowability at high temperature, which can fully fill the triangular microtexture of mold rollers. However, when the mold roller leaves the polymer film, collapse defects may occur due to insufficient cooling. Therefore, the optimal imprinting temperature for a high height-molding rate is selected as 85 °C.

(2) The effect of rolling speed on the height-molding rate of polymer film

**Figure 16:**
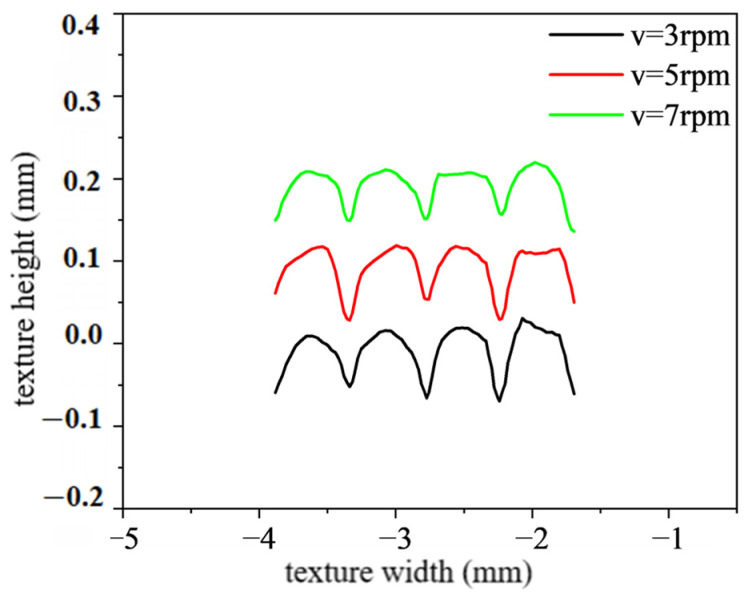
The texture shape under different rolling speed.

In this subsection, the effect of rolling speed on the height-molding rate of polymer film is analyzed, as shown in Figure 16. The rolling speeds are 3 rpm, 5 rpm and 7 rpm, respectively. The other parameters remain unchanged. It can be found that, under different rolling speeds, there is little difference in the width of surface texture of polymer films. But, there is a significant difference in the height of the surface texture of polymer films. The surface texture height of polymer films decreases with an increase in rolling speed. Specifically, when the rolling speeds are 3 rpm, 5 rpm and 7 rpm, the average height of the surface texture is 0.088 mm, 0.075 mm and 0.066 mm, respectively. This phenomenon is mainly due to the fact that the filling time of polymer materials decreases as the rolling speed of the roller increases. Due to the viscoelasticity of polymer materials at high temperatures, the filling time at high rolling speeds is insufficient to ensure that the polymer material can fully fill the triangular surface texture of the mold roller. In addition, considering appropriate production efficiency, the optimal rolling speed for a high height-molding rate is selected as 3 rpm.

(3) The effect of the surface texture size of the template on the height-molding rate of polymer film

In this subsection, the effect of the surface texture size of the template on the height-molding rate of polymer film is analyzed, as shown in Table 7, where the height-molding rate *P* (%) is the ratio of the height of the triangular texture formed by the actual rolling *h* (mm) to the height of the triangular texture on the mold roller *h*_0_ (mm) at the same rolling distance. The other parameters remain unchanged, with an imprinting temperature of 85 °C and a rolling speed of 3 rpm. It can be found that the surface texture size of the template has a significant effect on the height-molding rate of the surface texture of polymer films. The height-molding rate of polymer film ranges from 28.31% to 59.10%. The height-molding rate of the surface texture of polymer films decreases with an increase in the bottom edge length and height of the surface texture of the template. The volume within the triangular surface texture of the mold roller increases with its the bottom edge length and height. In a single surface texture, the more polymer filled, the worse the heat dissipation effect and the more severe the rebound during the demolding process. This fact will cause the surface texture of the polymer film to collapse, reducing the height-molding rate of the surface texture.

(6) $$P=\frac{h}{h_{0}}$$

#### 3.2.2. Surface Texture

(1) The results of micro/nano imprinting at different imprinting temperatures

During the continuous micro/nano imprinting process, the temperature of the polymer film surface cannot be directly controlled. In this experiment, the temperature of the polymer film surface is indirectly controlled by adjusting the mold temperature of the polymer film. The infrared temperature camera is used to measure the temperature of the polymer film surface during the micro/nano imprinting process. 

**Figure 17:**
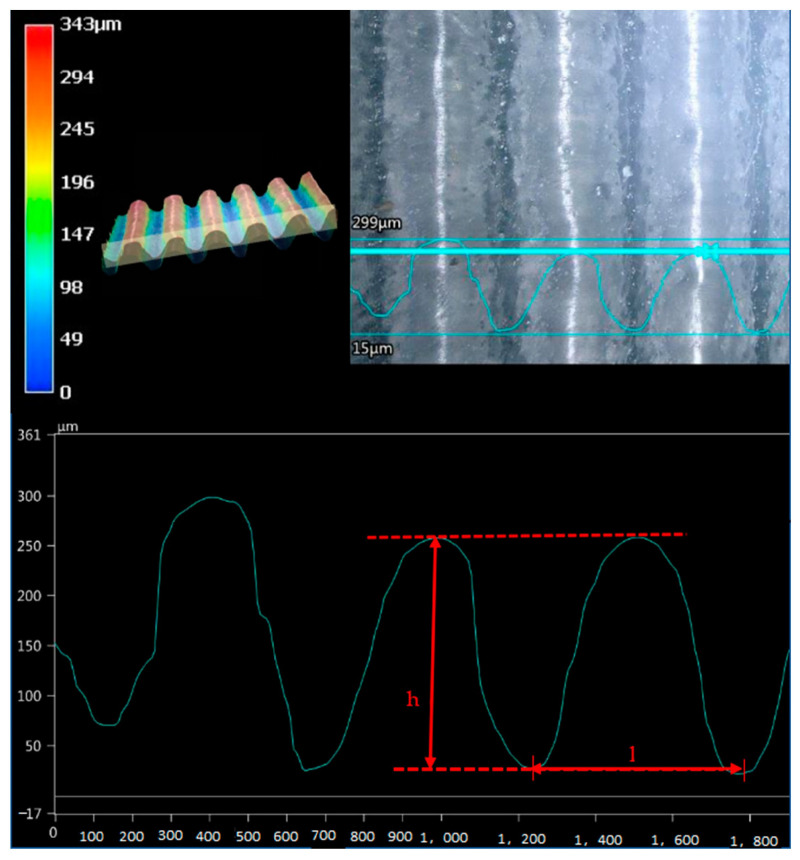
The measurement result of the surface texture height of polymer film.

**Figure 18:**
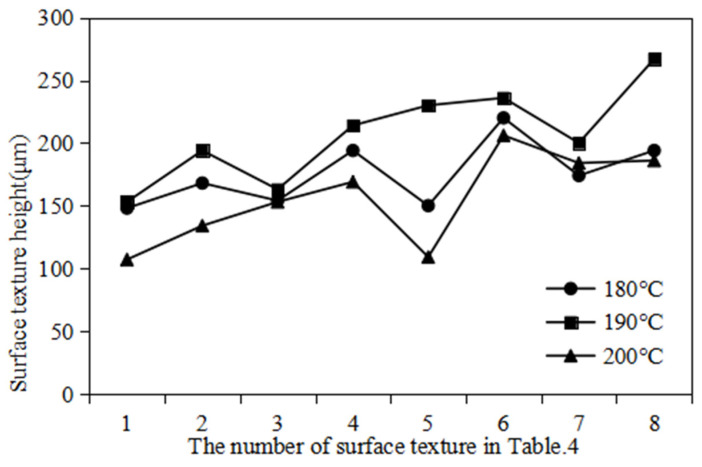
The surface texture height of polymer film at different imprinting temperatures.

Experimental measurements show that, when the mold temperatures are 180 °C, 190 °C, and 200 °C, the corresponding temperatures of the polymer film surface are 78.3 ± 4 °C, 85.2 ± 5 °C and 95.1 ± 5 °C, which are basically consistent with the temperature settings in the simulation model. Figure 17 shows the measurement result of the surface texture height of polymer film. Figure 18 shows the surface texture height of polymer film at different imprinting temperatures. It can be observed that, under the same surface texture size, the surface texture height is highest when the mold temperature is 190 °C. This phenomenon is consistent with the simulation results in the rheological filling model.

**Figure 19:**
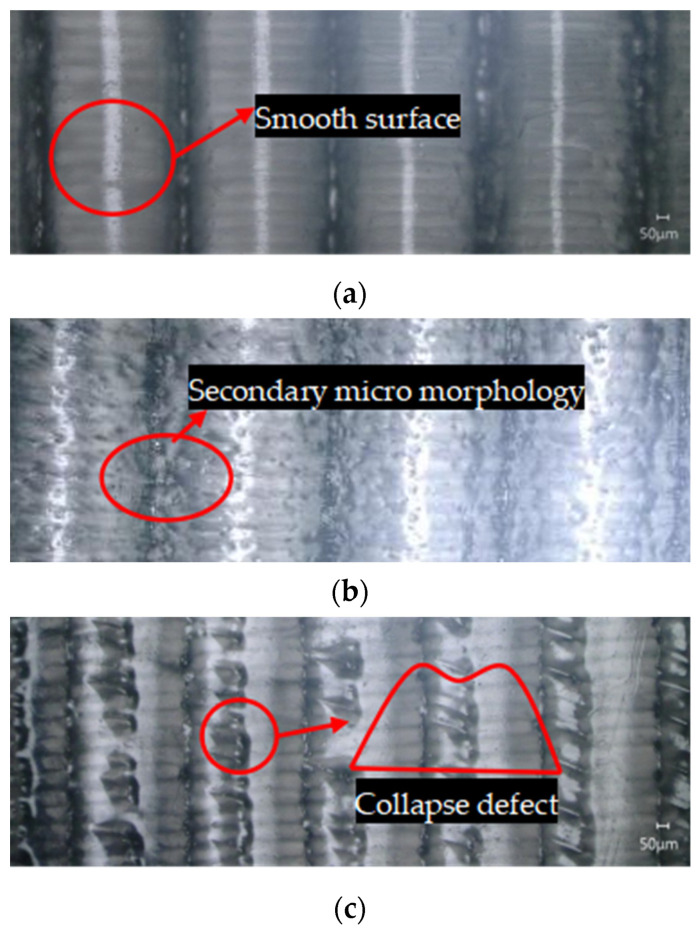
The surface texture micromorphology of polymer film at different imprinting temperatures. (**a**) 180 °C. (**b**) 190 °C. (**c**) 200 °C.

In addition, Figure 19 shows the surface texture micromorphology of polymer film at different imprinting temperatures. It can be observed that, when the mold temperature is 180 °C, the surface of the polymer film is relatively smooth. Almost no secondary microstructure can be seen. When the mold temperature is 200 °C, there are many collapse defects on the surface of the polymer film. When the mold temperature is 190 °C, many secondary microstructures and no collapse defects can be clearly observed on the surface of the polymer film. This phenomenon is mainly due to the fact that poor flowability of polymer material at low temperatures makes it difficult to replicate the discharge morphology from the mold rollers. Polymer material has good flowability at high temperatures, which can fully fill the triangular microtexture of mold rollers. However, when the mold roller leaves the polymer film, collapse defects may occur due to insufficient cooling.

Table 8 shows the height-molding rate of polymer film at different imprinting temperatures. It can be seen that, at the appropriate mold temperature (190 °C), all height-molding rates of the surface texture of the polymer film are higher than 60%. The highest height-molding rate is as high as 93.65%.

(2) The results of micro/nano imprinting at different rolling speeds

**Figure 20:**
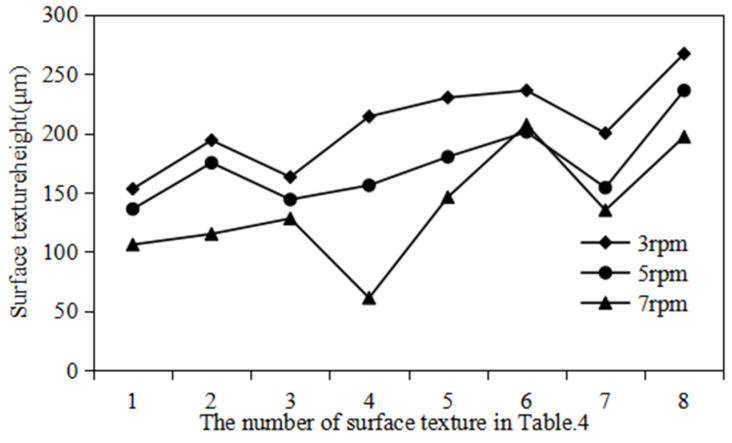
The surface texture height of polymer film at different rolling speeds.

**Figure 21:**
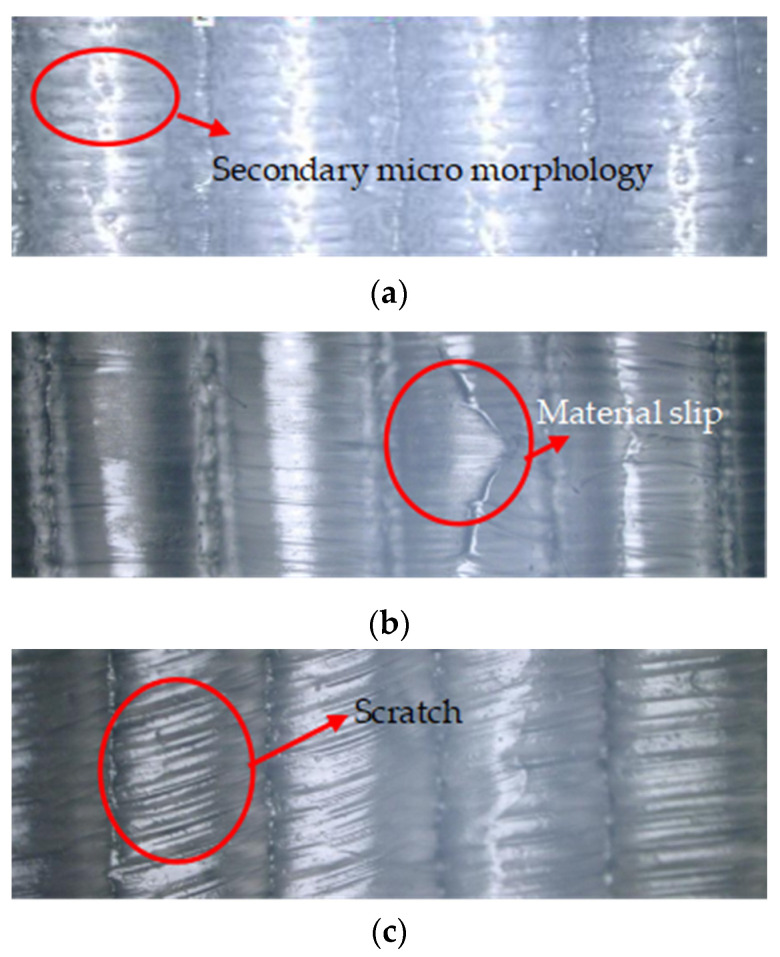
The surface texture micromorphology of polymer film at different rolling speeds. (**a**) 3 rpm. (**b**) 5 rpm. (**c**) 7 rpm.

Figure 20 shows the surface texture height of polymer film at different rolling speeds. It can be observed that, under the same surface texture size, the surface texture height decreases with an increase in the rolling speed. This phenomenon is consistent with the simulation results in the rheological filling model. In addition, Figure 21 shows the surface texture micromorphology of polymer film at different rolling speeds. This phenomenon is mainly due to the fact that, during the continuous micro/nano imprinting process, the polymer material filled in the surface texture of the mold roller is not cooled and solidified in time. Excessive rolling speed of the roller can lead to slippage or surface scratches of viscous polymer materials. Table 9 shows the height-molding rate of polymer film at different rolling speeds. It can be seen that, at the appropriate rolling speed (3 rpm), all height-molding rates of the surface texture of the polymer film are higher than 50%. The highest height-molding rate is as high as 93.65%.

(3) The results of micro/nano imprinting at different roller gaps

**Figure 22:**
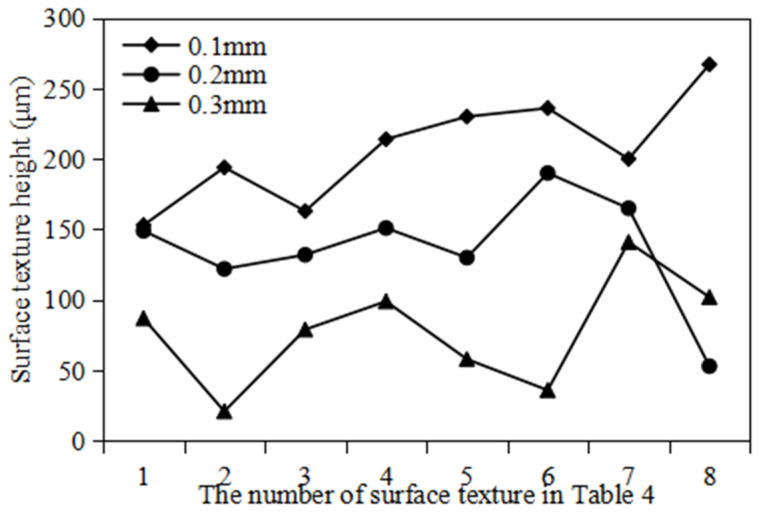
The surface texture height of polymer film at different roller gaps.

Figure 22 shows the surface texture height of polymer film at different roller gaps. It can be observed that, under the same surface texture size, the surface texture height decreases with an increase in the roller gap. This is mainly because small roller gaps can generate large pre-imprinting force. This is beneficial for the polymer material to fill the surface texture of the template roller. However, reducing the roller gap can also reduce the thickness of the polymer film. During the winding process, too small a thickness of polymer film may cause defects such as tensile deformation or fracture.

Table 10 shows the height-molding rate of polymer film at different roller gaps. It can be seen that, at the appropriate roller gap (0.1 mm), all height-molding rates of the surface texture of the polymer film are higher than 50%. The highest height-molding rate is as high as 93.65%.

Therefore, the optimal continuous micro–nano imprinting parameters are as follows: mold temperature of 190 °C, rolling speed of 3 rpm and roller gap of 0.1 mm. The highest height-molding rate of the polymer surface texture can reach 93.65%. In addition, when compared with Table 7, it can be found that the simulated height-molding rate is lower than the experimental height-molding rate, with a relative error of 6.9–12.1%. This is mainly because the actual height of the triangular surface texture is lower than the designed height of the surface texture. The specific reasons have been explained in Section 3.1.1. Low surface texture height and passivated triangular surface texture are beneficial for the filling of polymer materials. This indicates that the established rheological filling model of polymer material has high prediction accuracy for the height-molding rate.

#### 3.2.3. Solid–Liquid Contact Angle

(1) The polymer film with surface texture and without low surface energy coating

**Figure 23:**
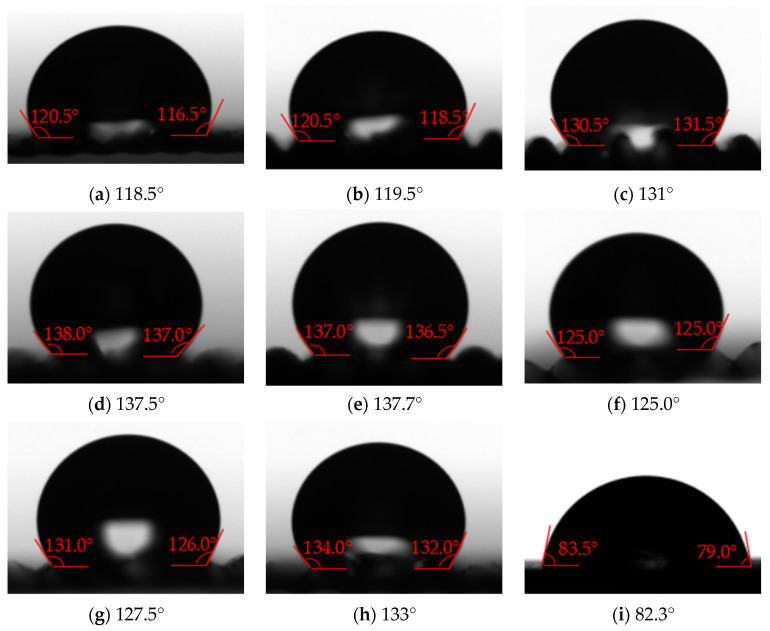
The measurement results of contact angle on the polymer film with surface texture and without low surface energy coating ((**a**–**h**) is No. 1–8 in Table 4, (**i**) without surface texture).

Figure 23 shows the measurement results of the contact angle on the polymer film with surface texture and without low surface energy coating. It can be seen that the apparent contact angle of the polymer film is 82.3°, which is a hydrophilic material. Constructing surface texture on the polymer film can effectively improve the solid–liquid contact angle, with the maximum solid–liquid contact angle of 137.7° and hydrophobicity. The size of the surface texture of polymer films has a significant impact on the solid–liquid contact angle. When the length of the bottom edge is the same, the solid–liquid contact angle increases with the height of the surface texture. A surface texture with a large ratio of height to bottom edge length can achieve a large solid–liquid contact angle. In addition, water droplets on the surface of the polymer film can come into contact with the bottom of the surface texture, and the solid–liquid contact state at this time is the Wenzel state.

(2) The polymer film without surface texture and with low surface energy coating

**Figure 24:**
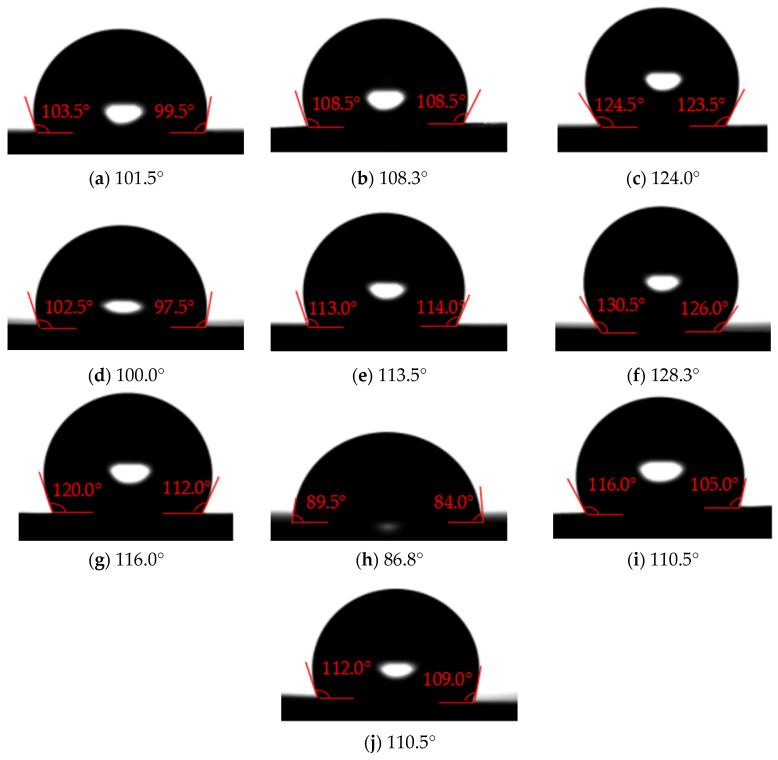
The measurement results of contact angle on the polymer film without surface texture and with low surface energy coating ((**a**–**j**) is No. 1–10 in Table 4).

The water contact angle of some films with surface texture can reach 130°, but the water adhesion on the surface of the polymer film is large, and the water droplets are difficult to roll off or slide off when the polymer film is tilted. In order to reduce the adhesion of the water droplets on the surface of the polymer film and further improve its hydrophobicity, SiO_2_-PDMS/butyl acetate dispersion suspension of different mass fractions is evenly sprayed onto the polymer film without surface texture. Figure 24 shows the measurement results of contact angle on the polymer film without surface texture and with low surface energy coating. It can be found that the low surface energy coating can effectively increase the solid–liquid contact angle on the polymer film. The maximum contact angle is 128.3° when the mass fractions are set as No. 6 in Figure 24.

(3) The polymer film with surface texture and with low surface energy coating

**Figure 25:**
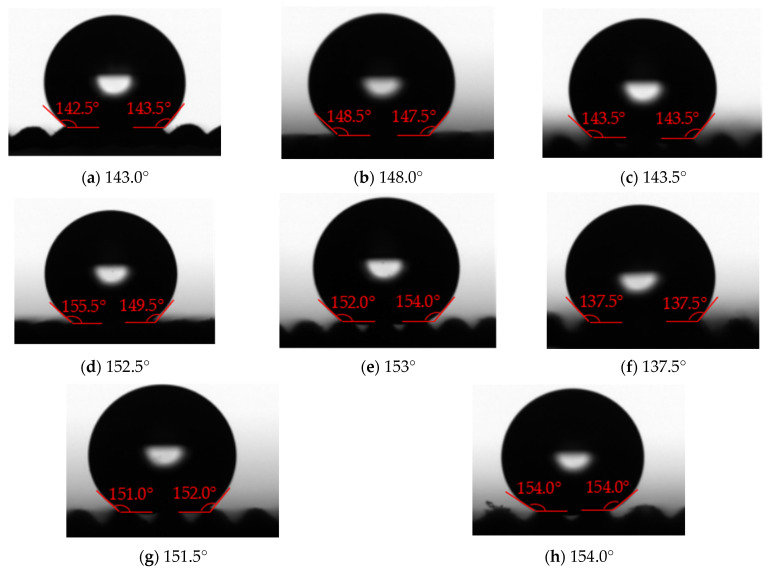
The measurement results of contact angle on the polymer film with surface texture and with low surface energy coating ((**a**–**h**) is No. 1–8 in Table 4).

SiO_2_-PDMS/butyl acetate dispersion suspension of No. 6 in Table 4 is selected in this experiment. Figure 25 shows the measurement results of contact angle on the polymer film with surface texture and with low surface energy coating. Compared with Figure 23, the low surface energy coating can obviously increase the solid–liquid contact angle by 12.5–28°. The range of the solid–liquid contact angle of polymer films is 137.5–154.0°. The solid–liquid contact angle and rolling angle on the surface of some polymer films are greater than 150° and less than 10°, respectively. Moreover, the solid–liquid contact angle increases with the height of the surface texture under the same bottom edge length. In Figure 25a–d,f, the solid–liquid contact state on the surface of polymer films is Wenzel state, while in Figure 25e,g,h, the solid–liquid contact state on the surface of polymer films is Cassie-Baxter state. In short, under the appropriate surface texture size and low surface energy coating, some polymer film surfaces exhibit superhydrophobic properties.

#### 3.2.4. Self-Cleaning Performance

**Figure 26:**
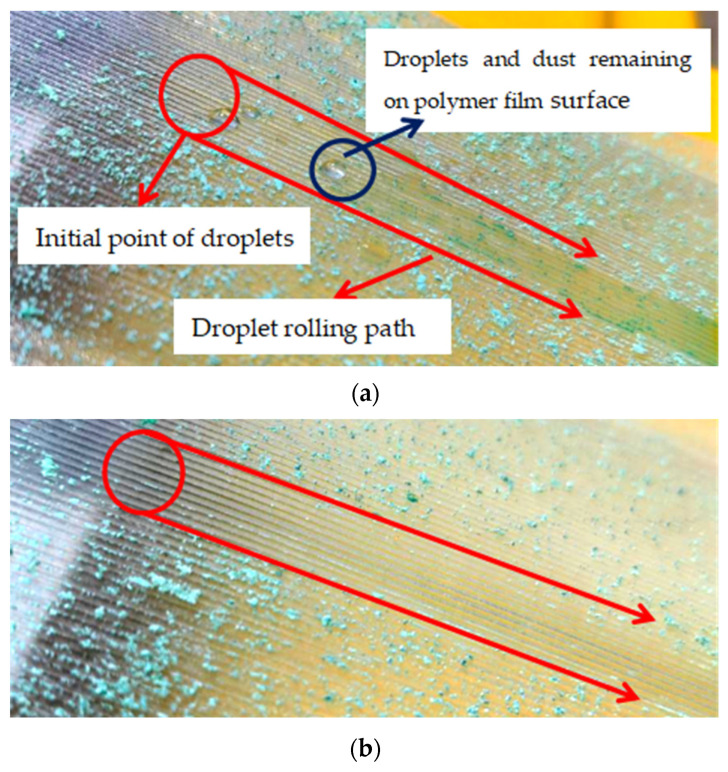
The results of a self-cleaning test on the polymer film surface. (**a**) Original polymer film. (**b**) Superhydrophobic polymer film.

Figure 26 shows the results of a self-cleaning test on the surface of polymer film. In Figure 26a shows the aftermath of water droplets flowing across the surface of the polymer film; some blue dust and small water droplets remain on the polymer film surface. It can be observed that the contact angle between residual water droplets and polymer film surface is small. This phenomenon is mainly due to the hydrophilicity of the original polymer film. In Figure 26b, after the water droplet has landed on the surface of the superhydrophobic polymer film, it undergoes a slight rebound and downward movement until it completely leaves the surface of the polymer film. On the water droplet’s rolling path, almost no blue dust or small water droplets are observed. This indicates that the superhydrophobic polymer film has good self-cleaning performance. Furthermore, polymer film is placed under the faucet and impacted by running water for 30 min. It can be found that the surface of the polymer film still has good self-cleaning properties.

#### 3.2.5. Anti-Icing Performance

**Figure 27:**
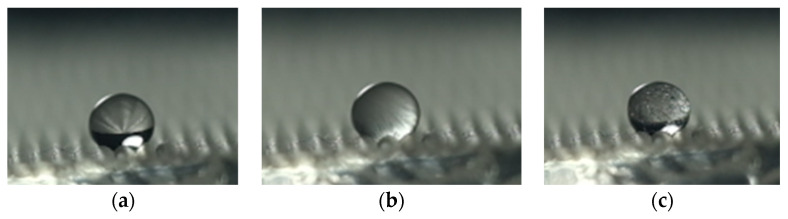
The freezing process of water droplets on the surface of polymer film. (**a**) Initial state. (**b**) Start freezing. (**c**) Complete freezing.

Figure 27 shows the freezing process of water droplets on the surface of polymer film. Table 11 shows the freezing times on different polymer film surfaces. The order of polymer films, based on their start freezing and complete freezing time, from long to short, is as follows: film without surface texture and coating, film with surface texture and without coating, and film with surface texture and coating. This is mainly because, when the film surface changes from hydrophilic surface to hydrophobic surface, the contact area between the water droplets and the polymer surface is reduced. This results in a decrease in the solid–liquid heat transfer area, thereby delaying the starting time of freezing. When the film surface transforms from hydrophobic surface to superhydrophobic surface, the solid–liquid contact state transforms from the Wenzel state to the Cassie–Baxter state. Due to the “air cushion effect”, the contact area between water droplets and film surfaces is further reduced, thereby further delaying the starting time of freezing. In addition, it can be observed that the time difference between complete freezing and start freezing on the surfaces of the three polymer films is basically the same. This is mainly because the density of solid water is smaller than that of liquid water, and the volume of frozen water droplets increases. This transforms the Cassie–Baxter state to the Wenzel state, increasing the contact area between the film surface and water droplets.

## 4. Conclusions

In this paper, a method of preparing superhydrophobic polymer film by roll-to-roll micro–nano imprinting is proposed. The mold roller is processed by wire electrical discharge machining to obtain a mold roller with a multi-level layered microstructure and good wear resistance. The optimum process parameters of rolling were obtained by simulation and experiment, and the polymer film with good hydrophobicity, self-cleaning and anti-icing properties was obtained.

(1) The simulation results of the rheological filling model show that the surface texture height of polymer films increases first and then decreases with an increase in the imprinting temperature, decreases with an increase in rolling speed, decreases with an increase in the bottom edge length and height of the surface texture of template. The optimal imprinting temperature and rolling speed for a high height-molding rate is selected as 85 °C and 3 rpm, respectively.
(2) After WEDM, the actual size of the surface texture of the template roller is basically the same as the design size. There are many anisotropic micro/submicron pits and protrusions distributed on the template surface. This discharge topography can provide basic conditions for the preparation of hierarchical microstructures. After 250 times of grinding, the contact angle on the template surface of the superhydrophobic sample still exceeds 150°.
(3) The optimal parameters obtained from experimental research on the influence of process parameters in continuous micro/nano imprinting are consistent with the simulation model. This indicates that the established rheological filling model has good guiding value.
(4) The maximum contact angle of the polymer film is 154°, which has good self-cleaning and anti-icing performance.

In the future, the prepared superhydrophobic thin films will need to undergo engineering tests such as acid rain, strong ultraviolet radiation and high-temperature aging before they can be applied to the surface of solar cells or other fields.

## Figures and Tables

**Table 1:** The physical parameters of low-density polyethylene.

Physical Parameters	Melt-Flow Rate	Density	Tensile Strengthening Strength	Tensile Breaking Strength	Tensile Yield Strength	Breaking Elongation
Value/Unit	2 g/10 min	920 kg/m^3^	28 MPa	36 MPa	10 MPa	800%

**Table 2:** The physical parameters of nano silica.

Parameters	Average Particle Size	Specific Surface Area	Volume Density	Density	Crystal Form
Value	20 nm	230 m^2^/g	0.06 g/cm^3^	2.4 g/cm^3^	Spherical

**Table 3:** The composition design of the SiO_2_-PDMS/butyl acetate dispersion suspension.

No.	SiO_2_ Content (g)	PDMS Content (g)	Butyl Acetate Solution (mL)
1	0.30	1.5	30
2	0.75	1.5	30
3	0.90	1.5	30
4	0.30	2.4	30
5	0.60	2.4	30
6	0.75	2.4	30
7	0.90	2.4	30
8	0.30	3.0	30
9	0.60	3.0	30
10	0.90	3.0	30

**Table 4:** The parameter design of the triangular surface texture on the roller template.

No.	Bottom Edge Length l (mm)	Height h (mm)
1	0.55	0.30
2	0.55	0.35
3	0.6	0.30
4	0.6	0.35
5	0.6	0.40
6	0.7	0.30
7	0.7	0.35
8	0.7	0.45

**Table 5:** The actual structural size of the triangular surface texture on the roller template.

	Actual Structural Size		Designed Structural Size	
No.	Bottom Edge Length l (mm)	Height h (mm)	Bottom Edge Length l (mm)	Height h (mm)
1	0.538	0.233	0.55	0.30
2	0.560	0.321	0.55	0.35
3	0.605	0.244	0.6	0.30
4	0.585	0.332	0.6	0.35
5	0.615	0.384	0.6	0.40
6	0.709	0.252	0.7	0.30
7	0.730	0.302	0.7	0.35
8	0.719	0.421	0.7	0.45

**Table 6:** The wear resistance of superhydrophobic samples prepared by different methods.

No.	Preparation Method	Sandpaper Mesh Number (#)	Pressure (kPa)	Grinding Length (mm)
1	Nanosecond laser [26]	600	10.0	250
2	Chemical precipitation method [27]	400	4.7	700
3	Spray coating method [28]	800	2.0	4800
4	Electroplating and fluorination treatment [29]	1000	4.5	1500
5	Electroplating method [30]	1000	1.6	2000
6	WEDM	240	62	12,500

**Table 7:** The effect of the surface texture size of the template on the height-molding rate of polymer film.

No.	The Surface Texture Size of Template		Height Molding Rate (%)
	Bottom Edge Length (mm)	Height (mm)	
1	0.55	0.25	59.71
2	0.55	0.30	55.12
3	0.60	0.25	59.73
4	0.60	0.30	58.67
5	0.70	0.25	53.45
6	0.70	0.30	84.3
7	0.7	0.35	61.2
8	0.7	0.45	59.3

**Table 8:** The height-molding rate (%) of polymer film at different imprinting temperatures.

No.	1	2	3	4	5	6	7	8
180 °C	63.52	52.34	63.11	58.43	39.06	87.3	57.62	46.08
190 °C	65.67	60.44	66.8	64.46	59.9	93.65	66.23	63.42
200 °C	45.92	41.74	62.7	50.9	28.39	81.75	60.93	44.18

**Table 9:** The height-molding rate (%) of polymer film at different rolling speeds.

No.	1	2	3	4	5	6	7	8
3 rpm	65.67	60.44	66.8	64.46	59.9	93.65	66.23	63.42
5 rpm	58.37	54.52	59.02	46.99	46.88	79.76	50.99	56.06
7 rpm	45.49	35.83	52.46	18.37	38.02	82.14	44.7	46.79

**Table 10:** The height-molding rate of polymer film at different roller gaps.

No.	1	2	3	4	5	6	7	8
0.1 mm	65.67	60.44	66.8	64.46	59.9	93.65	66.23	63.42
0.2 mm	63.95	38.01	54.1	45.48	33.85	75.4	54.64	12.59
0.3 mm	37.34	6.54	32.38	29.82	15.1	14.29	46.69	24.23

**Table 11:** The freezing times on different polymer film surfaces.

	Without Surface Texture and Coating	With Surface Texture and without Coating	With Surface Texture and Coating
Start freezing(s)	125	502	1025
Complete freezing(s)	197	576	1080

## Data Availability

No new data were created or analyzed in this study. Data sharing is not applicable to this article.
